# Facial Expression Recognition with LBP and ORB Features

**DOI:** 10.1155/2021/8828245

**Published:** 2021-01-12

**Authors:** Ben Niu, Zhenxing Gao, Bingbing Guo

**Affiliations:** ^1^School of Electronic and Information Engineering, Jinling Institute of Technology, Nanjing 211169, China; ^2^College of Civil Aviation, Nanjing University of Aeronautics and Astronautics, Nanjing 210016, China; ^3^School of Psychology, South China Normal University, Guangzhou 510631, China

## Abstract

Emotion plays an important role in communication. For human–computer interaction, facial expression recognition has become an indispensable part. Recently, deep neural networks (DNNs) are widely used in this field and they overcome the limitations of conventional approaches. However, application of DNNs is very limited due to excessive hardware specifications requirement. Considering low hardware specifications used in real-life conditions, to gain better results without DNNs, in this paper, we propose an algorithm with the combination of the oriented FAST and rotated BRIEF (ORB) features and Local Binary Patterns (LBP) features extracted from facial expression. First of all, every image is passed through face detection algorithm to extract more effective features. Second, in order to increase computational speed, the ORB and LBP features are extracted from the face region; specifically, region division is innovatively employed in the traditional ORB to avoid the concentration of the features. The features are invariant to scale and grayscale as well as rotation changes. Finally, the combined features are classified by Support Vector Machine (SVM). The proposed method is evaluated on several challenging databases such as Cohn-Kanade database (CK+), Japanese Female Facial Expressions database (JAFFE), and MMI database; experimental results of seven emotion state (neutral, joy, sadness, surprise, anger, fear, and disgust) show that the proposed framework is effective and accurate.

## 1. Introduction

Emotion recognition plays an important role in communication and is still a challenging research field. Facial expression is an indicator of feelings, providing valuable emotional information of human [1, 2]. Therefore, people can immediately recognize the emotional state of another person based on his/her facial expression. Consequently, information on facial expressions is often used in automatic systems of emotion recognition. With the development of technology, it has become convenient for us to solve problems with automatic systems, such as recognizing a person's emotion from a facial image. A machine can accurately interact with humans if it has the ability to recognize human emotions. The real-world applications that involve such interactions include human–computer interaction (HCI) [3, 4], virtual reality (VR) [5], augmented reality (AR) [6], advanced driver assistant systems (ADASs) [7], and entertainment [8, 9]. For facial expression recognition system, faces are detected and recorded as 2D images by using various devices, such as electromyographs (EMGs), electrocardiographs (ECGs), electroencephalographs (EEG), and cameras.

Based on these 2D images, there are three main research directions in facial expression recognition. The first approach defines the movement of facial muscles as an action unit (AU), and the facial expression is represented by the movements of several AUs. According to the movements of AUs from an input image, the corresponding facial expression can be determined by decoding the detected AUs. However, since muscle movements are often slight, detecting AUs has accurate problems [10], which have severely restricted the development of this approach. The second approach is feature-based methods, which include three steps. In the first stage, the face is detected from an input image, and landmarks are identified in the face area. In the second stage, features are extracted from the face region by using Histogram of Oriented Gradient (HOG), LBP, and Gabor wavelet methods. In the third stage, facial expressions are finally recognized and classified by using classifiers such as SVMs, AdaBoost, and random forests. The third approach is DNN-based methods, which perform very well in facial expression recognition. Different from conventional approaches, a feature map of the input image is produced through filter collection in the convolution layers [11–13].

Although DNN-based methods generally perform better than conventional methods, they have problems related to the processing time and memory consumption. Considering low hardware specifications used in some real-life conditions, to gain better results without DNNs, we aim to recognize seven basic emotional states (neutral, happy, surprise, sadness, fear, anger, and disgust) based on facial expressions using the second (feature based) approach. As we knew, the LBP is a powerful feature for texture representation. When facial expression appears, different textures are generated by the actions of the related muscles on the face. For this reason, we chose LBP in our study for facial expression recognition. In addition, to achieve the requirements of real time that are usually overlooked in previous studies, we combine LBP and OBR, since the ORB has been shown to have high computing speed.

However, the traditional ORB is more concentrated on the distribution of the extracted feature points. As traditional ORB, abundant feature points are extracted to achieve a higher accuracy, but the excessively dense feature points are very inconvenient for feature description. To solve this problem, we innovatively employed region division in the traditional ORB. This improved ORB algorithm calculates the number of feature points extracted from each region according to the total number of feature points to be extracted and the number of regions to be divided. In the present study, we did not just combine LBP and ORB, but combined LBP with improved ORB which solved the problem of feature point overlap and redundancy in the extraction process. Moreover, to test our improved LBP and ORB algorithm, we conducted experiments on several facial expressions in CK+ database, JAFFE database, and MMI database.

This paper is organized as follows.  Section 2 summarizes some of the previous works.  Section 3 presents the algorithm framework of facial expression recognition. Faces are detected and extracted using the Dlib library because of its fast processing speed, and LBP and improved ORB features are extracted separately from the face region. Then, the *Z*-score method is used to fuse both types of features. The facial expression databases are given in Section 4, and the experimental results and comparison with state-of-the-art methods are presented in Section 5. Finally, conclusions are stated in Section 6.

## 2. Related Work

We describe related works of facial expression recognition systems that have been studied to date. These algorithms can largely be divided into three directions: the geometric feature extraction method, the appearance feature extraction method, and the deep learning-based method. The geometric feature extraction method extracts geometric elements of the facial structure and motion of the facial muscles, the appearance feature extraction method extracts the features of the entire facial criterion, and the deep learning-based method uses convolutional neural network (CNN) to achieve the automatic learning of the extracted facial features. Some of the recent algorithms involving the aforementioned algorithms are described in the following subsections.

The geometric features algorithm extracts the temporal or dynamic changes of the landmark of the face. Geometric relationship between landmark points which are detected from face regions are exploited for expression representation. Tang et al. designed geometric features based on psychology and physiology [14]. Liang et al. focused on fine-grained facial expression recognition in the wild and built a brand-new benchmark FG-Emotions, extended the original six classes to more elaborate thirty-three classes, and proposed a new end-to-end Multiscale Action Unit- (AU-) Based Network (MSAU-Net) for facial expression recognition [15]. Zhang et al. proposed an end-to-end deep learning model that allows to synthesize simultaneous facial images and pose-invariant facial expression recognition by exploiting shape geometry of the face image [16]. However, when the landmark points are detected incorrectly, the recognition accuracies will be decreased.

The appearance features are extracted from facial image intensities to represent a discriminative textural pattern. Mandal et al. extracted the adaptive positional thresholds for a facial image; threshold parameters in the local neighborhood can be adaptively adjusted for different images; then multidistance magnitude features are encoded. SVM is used as a classifier for the facial expression recognition [17]. Tsai combined the Haar-like features method with the self-quotient image (SQI) filter for facial expression recognition. The angular radial transform (ART), the discrete cosine transform (DCT), and the Gabor filter (GF) are simultaneously employed in the system. An SVM is used to achieve the best results [18]. Chen et al. used visual modalities (face images) and audio modalities (speech) for facial expression recognition. They proposed a descriptor named Histogram of Oriented Gradients from Three Orthogonal Planes (HOG-TOP) to extract dynamic textures from video sequences to characterize facial appearance changes [19]. Ryu et al. used a coarse grid for stable codes (highly related to nonexpression), and a finer one for active codes (highly related to expression) as the features for facial expression recognition [20]. Liu et al. extracted LBP and HOG features from the salient areas that were defined on the faces; then Principal Component Analysis (PCA) is used to reduce the dimensions of the features which fused with LBP and HOG [21]. Ghimire et al. divided the whole face region into domain-specific local regions; then region-specific appearance features and geometric features are extracted from the domain-specific regions for facial expression recognition [22]. Nigam et al. retrieved Histogram of Oriented Gradients (HOG) feature in DWT domain and an SVM was used for expression recognition [23]. Kamarol et al. proposed spatiotemporal texture map (STTM) to capture subtle spatial and temporal variations of facial expressions with low computational complexity. And an SVM was used for classification [24]. Turan and Lam performed 27 local descriptors while the Local Phase Quantization (LPQ) and Local Gabor Binary Pattern Histogram Sequence (LGBPHS) achieved the best results [25]. Bougourzi et al. combined histogram of oriented gradients, local phase quantization, and binarized statistical image features to recognize the facial expressions from the static images [26]. Meena et al. used graph signal processing along with the curvelet transform for recognizing the facial expressions. Not only the dimension of the feature vectors has been reduced but also recognition of the facial expression has been significantly improved [27]. Ashir et al. used the compressive sensing technique with statistical analysis of the extracted compressed facial signal to represent a more robust feature representation for each individual facial expression class [28].

Recently, deep learning is widely used in many computer vision tasks including facial expression recognition and achieves remarkable success. The facial expression features are learned automatically by Convolutional Neural Networks (CNN). Dong et al. introduced dense connectivity across pooling to enforce feature sharing in a relatively shallow CNN structure for effective representation of facial expressions under limited training data [29]. Xie et al. embedded the feature sparseness into deep feature learning to boost the generalization ability of the convolutional neural network for facial expression recognition [30]. Yu et al. designed a multitask learning framework for global–local representation of facial expressions [31]. Wen et al. designed a new neural network with domain information loss and dynamic objectives learning; the network can avoid the gradient disappearance and obtain the higher semantic features [32]. Wang et al. proposed a simple yet efficient Self-Cure Network (SCN) which suppresses the uncertainties efficiently and prevented deep networks from overfitting uncertain facial images [33]. Although deep learning based methodology may achieve higher recognition rate, more computing resources and data are needed. For these reasons, our research uses the former framework.

## 3. Methodology

**Figure 1:**
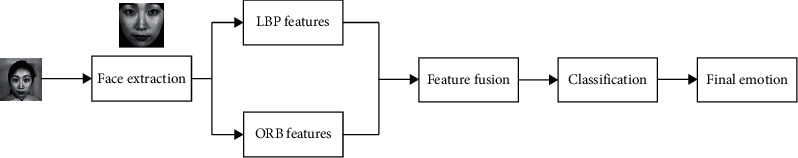
Flow diagram of emotion recognition.

In this section, the proposed methodology is introduced in detail. As shown in Figure 1, before LBP and ORB features extraction, the input images should be preprocessed (face detection and face extraction). The LBP is a fine-scale descriptor that captures small textural details. Local spatial invariance is achieved by locally pooling (e.g., with a histogram). Given that this approach is very resistant to lighting changes, LBP is a good choice for coding the fine details of facial appearance information over a range of coarse scales. In addition, ORB methods are 2 orders of magnitude faster than SIFT methods, and they perform as well in many situations. Moreover, our improved ORB avoided feature point overlap and redundancy in extraction processing. Then, features fusion of LBP and improved ORB were performed. Finally, by using the SVM, the classification accuracy was calculated to estimate whether our method can achieve outstanding results in facial expression recognition.

### 3.1. Face Detection and Face Extraction

**Figure 2:**
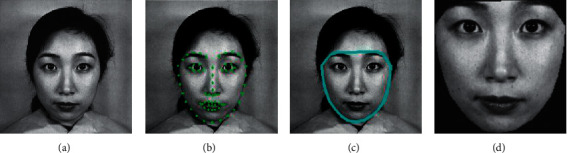
The framework of face detection and face extraction. (a) The original image. (b) The detected face with 68 landmarks. (c) The face region using points 1–27. (d) The extracted face region.

Many algorithms have been proposed for face detection, such as the Haar cascade algorithm, HOG method with an SVM, and CNN models. In this paper, we use the Dlib library to detect faces because of its fast processing speed as shown in Figure 2. The Dlib library contains a facial landmark detector with a pretrained model [34, 35]. This model marks 68 points on the face, and the points specify regions of the face. The jaw line is highlighted using points 1–17, the left eyebrow is highlighted using points 18–22, the right eyebrow is highlighted using points 23–27, the left eye region is highlighted using points 37–42, the right eye region is highlighted using points 43–48, the region of the nose is highlighted using points 28–36, the outer lip area is highlighted using points 49–60, and the inner lip structure is highlighted using points 61–68. In this method, a regression tree model is created to find these facial landmark points directly from the pixel intensities without feature extraction; thus, the detection process may be fast enough to overcome the accuracy and quality problems associated with real-time analysis.

Usually, emotions are mainly conveyed via the eyes, nose, eyebrows, and some facial regions, so other parts of the face, such as the ears and forehead, can be excluded in further analysis. This face detection algorithm is appropriate for obtaining exact facial regions, and points 1–27 are used to extract the face region from original images.

### 3.2. Fusion with LBP and ORB Features

#### 3.2.1. Local Binary Patterns (LBPs)

LBPs, which were introduced by Ojala, can effectively describe the texture information of an image [36–38]. The LBP operator is defined for 3 × 3 neighborhoods, takes each pixel as the central pixel, and assesses the 8 pixels around the selected pixel based on a given threshold. The resulting binary-valued image patch forms a local image descriptor [39]. The LBP operator takes the following form:

(1) $$\begin{array}{c} \text{LBP}\left(x_{c},y_{c}\right)=\sum_{n=0}^{7}2^{n}s\left(i_{n}-i_{c}\right). \end{array}$$

**Figure 3:**
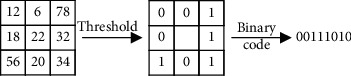
Original LBP operation.

**Figure 4:**
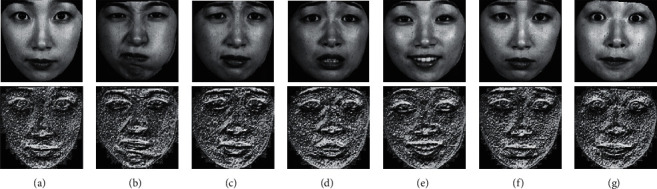
Original LBP features of facial expressions. (a) Neutral. (b) Anger. (c) Disgust. (d) Fear. (e) Happy. (f) Sad. (g) Surprise.

For the 8 neighbors of the central pixel *c*, *i*_*c*_ and *i*_*n*_ are the gray values at *c* and *n*, and *s*(*u*) is 1 if *u* ≥ 0 and 0 otherwise. An original LBP operator that is shown in Figures 3 and 4 depicts the original LBP features of facial expressions.

If an LBP operator contains at most one 0-1 and one 1-0 transition in a binary code, then a uniform pattern exists. The uniform pattern contains primitive structural information for edges and corners. This information can be used to reduce the length of the feature vector and implement a simple rotation-invariant descriptor. In our research, a uniform-pattern LBP descriptor is applied to obtain features from faces, and the length of the feature vector for a single cell can be reduced from 256 in the traditional method to 59. The size of the face region is 130 × 130, and the LBP face is 128 × 128, which is separated into small 16 × 16 patches with a resolution of 8 × 8. The uniform LBP features are extracted from each small patch and mapped to a 59-dimensional histogram.

#### 3.2.2. Oriented FAST and Rotated BRIEF (ORB) Features

FAST features are widely used because of their computational properties. The FAST operation uses one parameter, the intensity threshold between the center pixel and each pixel in circular ring around the center, as a simple but effective measure of corner orientation. This parameter is called the intensity centroid. The intensity centroid assumes that the intensity of a corner is different from that of a pixel center, and this vector may be used to determine an orientation [40].

*(1) FAST Detector*. The FAST detector first proposed by Rosten is widely used in corner detection for computer vision because of its rapid operations and low computational complexity compared to other corner detectors. The segment test criterion is based on analyzing a circle of sixteen pixels around the candidate corner *p*. The original detector classifies *p* as a corner if a set of *n* contiguous pixels that are brighter than the intensity of the candidate corner *I*_*p*_ plus a threshold *t* or are darker than *I*_*p*_ minus *t* exists in the circle. Notably, *n* is chosen as 12 because it yields a high-speed test that can be used to exclude a very large number of noncorners. The expression of the FAST detector is presented as follows:

(2) $$\begin{array}{c} S_{p⟶x}=\left\{\begin{array}{cc} d, & I_{p⟶x}\leq I_{p}-t,\\ s, & I_{p}-t<I_{p⟶x}<I_{p}+t,\\ b, & I_{p}+t\leq I_{p⟶x}, \end{array}\right. \end{array}$$

where *I*_*p*_ is the intensity of *p*, *I*_*p*⟶*x*_ is the intensity of the sixteen pixels around the corner, and *t* is a threshold. If *S*_*p*⟶*x*_ is equal to *d*, the pixel belongs to the darker group; if *S*_*p*⟶*x*_ is equal to *s*, the pixel belongs to the similar group. If *S*_*p*⟶*x*_ is equal to *b*, the pixel belongs to the brighter group. If 12 continuous pixels that belong to the darker or brighter group exist, *p* is regarded as a corner.

The corners are determined when all image pixels are tested using the above process. The corners will converge in some areas. To find the most robust corners, a nonmaximal suppression method based on a score function is adopted. The score values of each detected corner are calculated, and the corners with the low score values are removed. The corners with high score values are kept using the nonmaximal suppression method. There are several intuitive definitions for the score value:

1. The maximum value of *n* for which *p* is still a corner,
2. The minimum value of *t* for which *p* is still a corner,
3. The sum of the absolute difference between pixels in a contiguous arc and the center pixel.

Definitions (1) and (2) are highly quantifiable measures, and many pixels share these same values. To improve the computational speed, a slightly modified version of (3) is used. The score value is calculated as follows:

(3) $$\begin{array}{c} \text{score}=\max\left(\sum_{x\in S_{\text{bright}}}\left|I_{p⟶x}-I_{p}\right|-t,\sum_{x\in S_{\text{dark}}}\left|I_{p}-I_{p⟶x}\right|-t\right), \end{array}$$

where *I*_*p*_ is the intensity of *p*, *I*_*p*⟶*x*_ is the intensity of the sixteen pixels around the corner, and *t* is a threshold.

*(2) BRIEF Descriptor*. The BRIEF descriptor first proposed by M. Calonder is adopted to describe the detected corners. The form of the BRIEF descriptor consists of “1” and “0” values, and the length of the BRIEF descriptor is generally defined as 128 bits, 256 bits, or 512 bits. The following formula clearly shows the definition of the BRIEF descriptor:

(4) $$\begin{array}{c} \lambda \left(p;r_{1},c_{1},r_{2},c_{2}\right)=\left\{\begin{array}{c} 1:I\left(r_{1},c_{1}\right)<I\left(r_{2},c_{2}\right),\\ 0:I\left(r_{1},c_{1}\right)\geq I\left(r_{2},c_{2}\right), \end{array}\right. \end{array}$$

where *I*(*r*_1_, *c*_1_) and *I*(*r*_2_, *c*_2_) are the intensities of the pixels at (*r*_1_, *c*_1_) and (*r*_2_, *c*_2_), respectively. If *I*(*r*_1_, *c*_1_) is less than *I*(*r*_2_, *c*_2_), then *λ*=1; otherwise, *λ*=0. The length of *λ* is designated as 256 bits in this paper.

**Figure 5:**
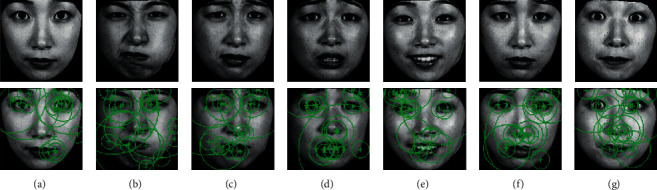
ORB features of facial expressions. (a) Neutral. (b) Anger. (c) Disgust. (d) Fear. (e) Happy. (f) Sad. (g) Surprise.

In the description of one point, a subimage with a size of 35 columns × 35 rows (here, the definition in the following situation is the same) is used. Because the BRIEF descriptor is sensitive to noise, the intensity value of each patch pair is calculated using a smoothing filter with a 5 × 5 subwindow centered at (*r*_*i*_, *c*_*i*_), where (*i*=1,2, Λ, 512). To reduce the impact of the image boundary, the intensity values of the image boundary are removed from the computation; thus, the actual size of the subimage is reduced to 31 × 31. Next, {(*r*_1_, *c*_1_), (*r*_2_, *c*_2_)} is defined as a patch pair instead of a point pair, and there is a total of 256 patch pairs in the subimage. The locations (*r*_*i*_, *c*_*i*_) of the 256 point pairs are determined by a Gaussian distribution, where (*r*_*i*_, *c*_*i*_) ~ *i*.*i*.*d*. Additionally, Gaussian (0, *S*^2^/25) : (*r*_*i*_, *c*_*i*_) values are determined from an isotropic Gaussian distribution, where *S* is the size of a patch. Details regarding how to determine the locations of the 256 patch pairs can be found in [40].  Figure 5 depicts the ORB features of facial expressions.

#### 3.2.3. Region-Based ORB

Traditional ORB is more concentrated on the distribution of the extracted feature points. In traditional ORB, abundant feature points are extracted to achieve a higher accuracy, but the excessively dense feature points are very inconvenient for feature description. To solve this problem, we innovatively employed region division in the traditional ORB. This improved ORB algorithm calculates the number of feature points to be extracted for each region according to the total number of feature points to be extracted and the number of regions to be divided. The steps are described as follows.

*Step 1*. Evenly divide the images into*M* × *N*regions of the same size. *M* is the row and *N* is the column of the division. Feature points are randomly distributed in the divided regions, and the regions are sequenced as{*h*_1_, *h*_2_,…, *h*_*M*×*N*_}.
*Step 2*. Set a threshold *T*
  (5) $$\begin{array}{c} T=\frac{n}{MN}, \end{array}$$
where *n* is the number of feature points.
*Step 3*. The feature points are detected in each region; if the number of features is not less than *T* , we choose *T*as the feature number. If the number of features is less than *T*, the threshold should be smaller and repeat this step.
*Step 4*. When the number of feature points is bigger than *n*, nonmaximal suppression method is used to select the best feature points.
*Step 5*. All regions are traversed until the number of feature points meets the conditions.

#### 3.2.4. Feature Fusion Scheme

Feature normalization is used to increase the recognition rate before feature fusion. In this article, the LBP features are normalized to the range of 0-1, and the following formula is applied to normalize the LBP and ORB features:

(6) $$\begin{array}{c} L=\frac{l}{\max\left(l\right)}, \end{array}$$

where *l* is the value of a feature.

Feature fusion is usually performed because one type of feature cannot describe every image characteristic. In this article, the LBP and ORB descriptors are fused by using the *Z*-score method.

(7) $$\begin{array}{c} \sigma =\sum_{j}^{J}{\left(f_{j}-\mu \right)}^{2},\\ \mu =\frac{\sum_{j}^{J}f_{j}}{J},\\ {\hat{f}}_{j}=K\frac{\left(x_{j}-\mu \right)}{\sigma +C}, \end{array}$$

where *f*_*j*_ is an LBP feature or ORB feature and ${\hat{f}}_{j}$ is the feature data after fusion. *K* is a factor multiplied by ${\hat{f}}_{j}$, and *K* is 100 in the following experiments.

## 4. Database of Facial Expressions

### 4.1. Japanese Female Facial Expressions (JAFFE) Database

**Figure 6:**
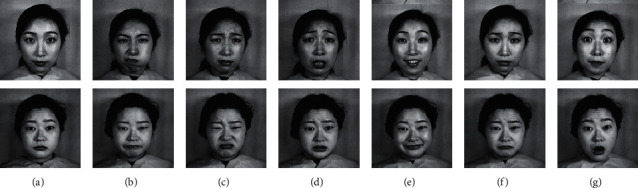
Some images from the JAFFE database. (a) Neutral. (b) Anger. (c) Disgust. (d) Fear. (e) Happy. (f) Sad. (g) Surprise.

The JAFFE database contains 213 images from ten different Japanese female subjects, and each subject has 3 or 4 examples of seven facial emotions (six basic facial emotions and one neutral emotion). The images are gray with a resolution of 256 × 256 [41]. In our experiment, the total 213 images (anger: 30 images; disgust: 29 images; fear: 32 images; happiness: 31 images; neutral: 30 images; sad: 31 images; and surprise: 30 images) are used to evaluate the proposed algorithm. Some images from the JAFFE database are shown in Figure 6.

### 4.2. The Extended Cohn-Kanade (CK+) Database

The CK+ database contains 593 sequences from 123 subjects from 18 to 30 years of age. Overall, 327 sequences are labeled with anger (45), contempt (18), disgust (59), fear (25), happiness (69), sad (28), and surprise (83). Different facial expressions have different numbers in the corresponding sequences, and each expression contains images from neutral to the peak expression. The neutral and three peak frames sampled from each sequence are used for testing while contempt is not considered. In our experiments, we selected 309 sequences which are labeled as one of the six basic facial expressions excluding contempt. We chose the three last frames of each sequence, so there were 927 images for anger, disgust, fear, happiness, sad, and surprise. In addition, we defined the first frame of each sequence as neutral facial expression (309 images). Taken together, 1236 images from CK+ database were used in our experiments. The images have resolution of 640 × 490 or 640 × 480 [42, 43].

### 4.3. MMI Database

**Figure 7:**
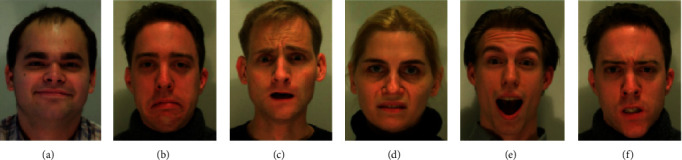
Some images from the MMI database.

The MMI facial expression database [44, 45] includes 208 videos of both genders aged from 19 to 62 years. Each sequence labels as one of the six basic emotions and begins with the neutral expression and ends with it, while the expression is in the middle. In our experiments, we approximated the three peak frames from the middle of each sequence. Thus, we obtained 624 color images in total with the resolution 720 × 576 pixels. The MMI database has more challenging conditions including illumination, gender, age, ethnicities, and insufficient number of subjects, of which many wear accessories (e.g., glasses, moustache).  Figure 7 shows some images from the MMI database.

In the collection of the static image from the sequences, the most commonly used method is to select the first frame (as the neutral expression) and the three-peak frame in each sequence. Also, ten-fold person-independent cross-validation is conducted for the experiments.

## 5. Experiments

This section gives the details of the experiments performed. Two experiments involving the JAFFE, CK+, and MMI databases were conducted to verify the effectiveness of the proposed facial expression recognition method. In the first experiment, the subjects used for training were part of the testing set. In the second experiment, the subjects used for training were not used for testing. That means subject-dependent (SD) and subject-independent (SI), and cross-validation schemes are used in Experiment 1 and Experiment 2, respectively. The subject-independent experimental scheme has been widely investigated in the past years because it is more plausible for the real applications, which need to recognize the facial expressions from new persons. An SVM was the basic classifier used in the experiments, and 10-fold cross-validation procedure was used in all experiments.

### 5.1. Experiments Based on the JAFFE Database

All the facial region images were resized to 128 × 128 pixels, and the classifiers were trained using LibSVM [46]. The parameters were defined through a grid search, and 10-fold cross-validation was used to improve algorithm performance. Each experiment was repeated 10 times, and the results were reported as the average of these 10 replications. Fusion features were used in the proposed algorithm, and the input data were fused with LBP and ORB features.

The accuracy of experiments with different features is shown in Table 1. Results of LBP + ORB were better than the results of LBP/ORB in Experiment 1 (LBP + ORB: 92.4%; LBP: 86.7%; and ORB: 89.2%) and Experiment 2 (LBP + ORB: 88.5%; LBP: 65.4%; and ORB: 73.1%).

A comparison of our results with those of other methods from the literature is shown in Table 2. Recognition rate based on JAFFE dataset suggested the proposed method obtained higher accuracy (88.5%) than recent methods (73.24%, 55.71%, and 87.6%).

### 5.2. Experiments Based on the CK+ Database

The procedure for the experiments based on the CK+ database was the same as that for the JAFFE database. All facial expression images were extracted from video sequences. The accuracy of experiments with different features is shown in Table 3. Results of LBP + ORB were better than the results of LBP/ORB in Experiment 1 (LBP + ORB: 99.2%; LBP: 97.3%; and ORB: 98.5%) and Experiment 2 (LBP + ORB: 93.2%; LBP: 86.7%; and ORB: 88.4%).

A comparison of our results with those of other methods from the literature is shown in Table 4. Recognition rate based on CK+ dataset suggested the proposed method obtained higher accuracy (93.2%) than one recent method (84.6%), but lower than three recent methods (95.96%, 94.5%, and 95.1%).

### 5.3. Experiments Based on the MMI Database

The procedure for the experiments based on the MMI database was the same as that for the JAFFE database and CK+ database. All facial expression images were extracted from video sequences. The accuracy of experiments with different features is shown in Table 5. Results of LBP + ORB were better than the results of LBP/ORB in Experiment 1 (LBP + ORB: 84.2%; LBP: 73.3%; and ORB: 78.5%) and Experiment 2 (LBP + ORB: 79.8%; LBP: 69.9%; and ORB: 73.3%).

A comparison of our results with those of other methods from the literature is shown in Table 6. The proposed method obtained higher accuracy (79.8%) than all four recent methods (73.57%, 70.63%, 76.3%, and 58.98%). Notably, the proposed method performed better than the other methods based on MMI dataset.

### 5.4. Discussion

Based on the results of our experiments, it is obvious that our proposed method can deal with the facial expression recognition efficiently. In this paper, we proposed a new framework of emotion recognition system from static images by using feature-based approach. We combined LBP and improved ORB features extracted by descriptors possessing different properties. Recently, numerous studies have reported that DNN-based approach performed well in facial expression recognition. However, DNN-based approach needs excessive hardware specifications requirement. Considering low hardware specifications used in real-life condition, to gain better results without DNNs, in this paper, we proposed an algorithm with the combination of the improved ORB features and LBP features extracted from facial expression.

Compared with other methods, LBP is more effective to extract facial expression. However, the speed is also important for effective facial expression recognition. Instead of using LBP only, ORB is also employed for facial expression recognition in our system since the ORB has been shown to have high computing speed. Notably, compared with LBP/ORB, experimental results based on JAFFE, CK+, and MMI databases showed that our method can enhance the accuracy of facial expression recognition, in other words, can improve the discriminative ability. Meanwhile, based on JAFFE, CK+, and MMI databases, we found that our method was in general better than recent studies on facial expression recognition. Our method improved the accuracy of facial expression recognition based on JAFFE database; results of recognition rate based on CK+ database indicated that our method was not as good as the previous methods but outperformed [17] which used similar experiment. Recognition rate based on MMI database suggested the proposed method achieved superior recognition accuracy than all four state-of-the-art approaches. Considering the characteristics of the facial expression and the requirements of real time, we combined LBP and improved OBR as the features of facial expression to improve recognition performance, but there are still much more challenges. In the future research, we will focus on how to improve the accuracy and increase the speed at the same time.

## 6. Conclusions

This paper proposed a framework for facial expression recognition with fused features. LBP and improved ORB descriptors were used for feature extraction, and SVM classification was performed for facial expression recognition. The experimental results showed that the proposed framework performed better than some widely used methods based on the JAFFE database, CK+ database, and MMI database.

This article mainly focused on facial expressions from static images. In our future work, facial expressions from video sequences will be further considered, as will calculation times for embedded systems.

## Figures and Tables

**Table 1:** The accuracy of experiments with different features.

Experiment 1		Experiment 2	
Features	Accuracy (%)	Features	Accuracy (%)
LBP	86.7	LBP	65.4
ORB	89.2	ORB	73.1
LBP + ORB	92.4	LBP + ORB	88.5

**Table 2:** Recognition rate based on JAFFE.

Study		Bougourzi et al. [26]	Mandal et al. [17]	Tong and Chen [47]	Proposed algorithm
Method		PCA-fusion	RADAP	LDDSCP	LBP + ORB
Validation setting		10-fold	10-fold	10-fold	10-fold
Accuracy (%)	SD	—	90.5	—	92.4
	SI	73.24	55.71	87.6	88.5

**Table 3:** The accuracy of experiments with different features.

Experiment 1		Experiment 2	
Features	Accuracy (%)	Features	Accuracy (%)
LBP	97.3	LBP	86.7
ORB	98.5	ORB	88.4
LBP + ORB	99.2	LBP + ORB	93.2

**Table 4:** Recognition rate based on CK+.

Study		Bougourzi et al. [26]	Makhmudkhujaev et al. [48]	Ji et al. [49]	Mandal et al. [17]	Proposed algorithm
Method		PCA-fusion	LDDP	ICID	RADAP	LBP + ORB
Validation setting		10-fold	—	—	10-fold	10-fold
Accuracy (%)	SD	—	—	—	95.4	99.2
	SI	95.96	94.5	95.1	84.6	93.2

**Table 5:** The accuracy of experiments with different features.

Experiment 1		Experiment 2	
Features	Accuracy (%)	Features	Accuracy (%)
LBP	73.3	LBP	69.9
ORB	78.5	ORB	73.3
LBP + ORB	84.2	LBP + ORB	79.8

**Table 6:** Recognition rate based on MMI.

Study		Bougourzi et al. [26]	Makhmudkhujaev et al. [48]	Ji et al. [49]	Mandal et al. [17]	Proposed algorithm
Method		PCA-fusion	LDDP	ICID	RADAP	LBP + ORB
Validation setting		10-fold	—	—	10-fold	10-fold
Accuracy (%)	SD	—	—	—	86.6	84.2
	SI	73.57	70.63	76.3	58.98	79.8

## Data Availability

The data used to support the findings of this study are available from the corresponding author upon reasonable request.
